# Risk factors of locoregional relapse in locally advanced breast cancer treated with neoadjuvant chemotherapy following mastectomy and radiotherapy

**DOI:** 10.18632/oncotarget.14407

**Published:** 2016-12-31

**Authors:** Liang Huang, Sheng Chen, Wentao T. Yang, Zhiming Shao

**Affiliations:** ^1^ Department of Breast Surgery, Fudan University Shanghai Cancer Center/Cancer Institute, Shanghai, China; ^2^ Department of Oncology, Shanghai Medical College, Fudan University, Shanghai, China; ^3^ Department of Pathology, Fudan University Shanghai Cancer Center/Cancer Institute, Shanghai, China

**Keywords:** neoadjuvant chemotherapy, non-pathological complete response, locoregional relapse, annual recurrence rate

## Abstract

We seek to investigate the prognostic factors that could possibly increase the locoregional recurrence of breast cancer patients who do not achieve pathological complete response after neoadjuvant chemotherapy, and to build a prognostic nomogram to predict patients' outcome. The retrospective analysis included 510 patients who had received neoadjuvant chemotherapy followed by surgery and radiotherapy. 62 locoregional events occurred after a median 61 months of follow-up. The five-year cumulative incidence of local recurrence and regional recurrence were 8.63% and 4.31%, respectively. Multivariate analysis revealed that positivity for ≥ 4 lymph nodes and Ki-67 index ≥ 14% were independent factors. According to our prognostic model, the 5-year locoregional free survival rates in the low, intermediate, and high-risk groups were 95.5%, 89.1%, and 67.1%, respectively (*p* < 0.001). Annual recurrence curves indicated that the relapse peak after mastectomy emerged in the first 1 year. Positivity for ≥ 4 lymph nodes and Ki-67 index ≥ 14% were independent factors for locoregional recurrence. This prognostic model has considerable clinical value in predicting locoregional recurrence, which could help clinicians to design appropriate locoregional treatment specifically and to perform surveillance individually.

## INTRODUCTION

Preoperative or neoadjuvant chemotherapy (NCT) is the standard treatment in locally advanced or inflammatory breast cancer [1]. In patients with operable breast cancer, neoadjuvant chemotherapy can allow increased rate of breast conservation surgery. The use of neoadjuvant chemotherapy has also provided insight into tumour biology and differential responses to treatment. Furthermore, pathologic complete response (pCR) is an early prognostic marker for better long-term outcome [2, 3].

When patients receive neoadjuvant chemotherapy, non-pCR status might be a high risk for relapse and metastasis [4]. Optimizing locoregional outcome is important, because these failures can ultimately lead to substantial morbidity, disease progression and death [5, 6]. Receiving NCT was associated with an increased risk of locoregional recurrence (LRR) compared with receiving adjuvant therapy [7]. Retrospective series have demonstrated that the elimination of radiotherapy after NCT in high-risk patients results in an unacceptably high rate of recurrence [8, 9]. Neoadjuvant chemotherapy followed by definitive surgery consolidated with postmastectomy radiation therapy has become the standard of care for patients with locally advantage breast cancer. It is clear that completion of this therapy is critical to locoregional control, but locoregional failure remains more common among patients with some molecular subtypes [10–12].

In contrast, there is limited information on the rates and predictors of LRR for patients who receive neoadjuvant chemotherapy, especially with the use of locoregional external radiotherapy after mastectomy. To address these questions, data from a single cancer institution provided us with the opportunity to examine the rates and patterns of LRR in patients treated with neoadjuvant chemotherapy and to identify independent predictors of LRR in this setting. In this study, we retrospectively analysed 510 neoadjuvant patients with residual tumour who completed mastectomy and postmastectomy radiation therapy (PMRT) in order to identify the independent prognostic factors and create a nomogram to distinguish patients with different outcomes.

## PATIENTS AND METHODS

### Study population

The study cohort in the present study was selected consecutively from patients with locally advanced breast cancer who had received NCT followed by surgery at Shanghai Cancer Centre from 1999 to 2011. The diagnoses were confirmed as invasive carcinoma by core needle biopsy, and node status was assessed by fine needle aspiration of palpable lymph nodes before NCT. After NCT, all patients underwent modified radical mastectomy and were confirmed to be non-pathological complete responders with residual tumour in the breast. Patients who underwent breast-conserving surgery or who did not undergo adjuvant radiotherapy were not eligible for this study. Other exclusion criteria included metastatic disease before surgery, bilateral breast cancer, male breast cancer, and inflammatory breast cancer.

We retrospectively reviewed a series of 510 patients with locally advanced breast cancer who met the above criteria. The NCT regimens included anthracycline-containing, vinorelbine-containing, and taxane-containing regimens for a median of 3 cycles, as previously reported [13]. Due to health insurance-related limitations, trastuzumab was not utilized before surgery in patients with HER2 overexpression. For all patients, the surgical procedure included mastectomy and axillary lymph node dissection upon completion of NCT. Additional cycles of chemotherapy were subsequently performed to complete a total of 6–8 cycles at the discretion of the treating physician. Radiation was delivered after completion of chemotherapy. The treatment volumes typically included the chest wall and draining lymphatics in the supraclavicular and infraclavicular nodal region (dose prescription was 50 Gy in 25 fractions). The internal nodal region was not routinely irradiated unless pathologically involved. For ER/PR-positive patients, adjuvant endocrine therapy was recommended. Local recurrence was defined as disease recurrence in the ipsilateral breast. Regional recurrence was defined as metastatic disease in the ipsilateral axillary, supraclavicular, infraclavicular or internal mammary lymph nodes. This study is a retrospective study without any type of clinical intervention. The study was conducted according to the principles expressed in the Declaration of Helsinki and approved by the institutional review board of Fudan University Shanghai Cancer Center. All the patients enrolled in this study signed the informed consent voluntarily.

### Treatment response

The clinical responses to neoadjuvant chemotherapy were evaluated based on MRI and ultrasound examinations and in accordance with the Response Evaluation Criteria in Solid Tumors (RECIST) 1.1 [14]. Two experienced pathologists evaluated samples for the presence of a pathological response. The Miller-Payne (MP) grading system was employed to evaluate the decrease in cancer cellularity [15]: No change or some alterations to individual malignant cells without a reduction in overall cellularity was classified as Grade 1; up to a 30% loss of tumour cells was classified as Grade 2; between an estimated 30% and 90% reduction in tumour cells was classified as Grade 3; more than a 90% loss of tumour cells with only small clusters or widely dispersed individual cells remaining was considered Grade 4; and no invasive malignant cells remaining was considered Grade 5. None of the patients enrolled in this study was considered a Grade 5 responder.

### Immunohistochemistry

Immunohistochemistry (IHC) analysis was performed on paraffin-embedded post-operative tissue sections using standard procedures for breast tumour specimens. The cut-off value for ER positivity and PR positivity was 1% positive tumour cells with nuclear staining. HER2 was evaluated as 0, 1+, 2+ or 3+ using circumferential membrane-bound staining; positivity (HER2+) was considered as 3+ using IHC or with positive florescent in situ hybridization (FISH), whereas cases with 0 to 1+ or 2+ without FISH detection were regarded as negative (HER2-). The Ki-67 value was expressed as the percentage of positive cells (at least 1000) with nuclear staining in each case. The following antibodies were used for IHC: ER (M7047, clone 1D5, Dako, Produktionsvej, Glostrup, Denmark), PR (M3569, clone PR 636, Dako), HER2 (A0485, polyclonal rabbit antibody, Dako), and Ki-67 (MIB-1, Dako).

### Literature search and literature-based data extraction

We searched the PubMed, Web of Science, and MEDLINE databases (updated to May 1, 2015) using the following search terms: “annual” and “recurrence” and “breast cancer.” Eligible studies and their references were retrieved and examined carefully. The literature inclusion criteria were as follows: (a) evaluation of ARR after surgery for primary breast cancer, (b) availability of information for ARR (numerical data or graphic data), and (c) full text published in English. Relevant information was carefully extracted from all eligible publications.

### Statistical analysis

Locoregional relapse-free survival (LRFS, defined as disease recurrence in the chest wall or regional lymph nodes) and distant relapse-free survival (DRFS, defined as distant disease metastasis) were calculated from the date of surgery to the date of disease relapse. Overall survival (OS) was calculated from the date of surgery to the date of death or last follow-up. Patients without relapse events or death were censored at the last follow-up. Multivariate analyses were performed with the Cox proportional model to determine the effects of independent prognostic factors. Survival curves were estimated using the Kaplan–Meier method, and the log-rank test was used to determine significance. All P-values reported are two-sided and were calculated at a significance level of 0.05. All statistical procedures were performed with SPSS 13.0 and STATA 11.0.

## RESULTS

We analyzed 510 breast cancer patients treated by neoadjuvant chemotherapy and underwent modified radical mastectomy followed by radiotherapy in Shanghai Cancer Centre between 1999 to 2011. These patients were divided by 4 groups based on the 2011 St. Gallen guidelines [16]. Table 1 lists the clinicopathological characteristics by constructed subtype. The median age of all patients was 49 years old (range 24-75 years). 74.5% of patients had clinically positive lymph nodes, and 43.3% of patients were in menopause. 96.1% of patients were diagnosed with invasive ductal carcinoma. Patients received two main regimens, including taxol-based regimens and non-taxol based regimens. There were no significant differences in the distribution of neoadjuvant regimens between the subtypes. The other clinicopathological features of patients were well-balanced. Overall, 60.6% (95% CI: 56.3%-64.9%) of the patients experienced a clinical objective response (CR or PR) as assessed by palpation, ultrasonography and/or MRI.

**Table 1:** Patients characteristics at baseline and clinical evaluation

	Luminal A *N*=202	Luminal B *N*=105	HER2+ *N*=62	TNBC *N*=141	*P* value
Age					0.232
≥50y	97	52	36	60	
<50y	105	53	26	81	
Menopause status					0.719
premenopause	115	61	31	82	
postmenopause	87	44	31	59	
cT stage					0.322
T2	66	35	15	47	
T3	98	56	35	79	
T4	38	14	12	15	
clinical N status					0.478
positive	143	80	48	109	
negative	59	25	14	32	
chemotherapy regimen					0.075
non-Taxel	129	68	42	108	
Taxel	73	37	20	33	
clinical response					0.360
CR/PR	131	63	37	78	
SD/PD	71	42	25	63	

After a median follow-up of 61 months (range 5-128 months), there were 40 isolated local recurrences, 17 isolated regional recurrences and 5 locoregional recurrences. Sixteen patients (25.8%) occurred synchronously with distant metastasis, and no LRR was detected after distant failure. Fifteen patients had recurrences in the supraclavicular field. The 5-year cumulative LRR-free survival rate was 88.0% (95% CI: 85.2%-90.1%). The 5-year overall survival and disease-free survival rates were 79.0% (75.5%-82.6%) and 63.1% (58.9%-67.3%), respectively. The luminal A subtype had the lowest local-region recurrence rate (6.44%), and the luminal B subtype had the highest LRR rate (22.86%). The distribution of local and regional recurrences by different molecular subtype was shown in Table 2. The cumulative incidence of local and regional recurrence was significantly greater in patients with the luminal B subtype (*p* < 0.001). The hormone receptor positive and HER2-negative subgroups had similar local and regional recurrences rate, whereas the other subgroups had a higher rate of local failure (*p* = 0.012).

**Table 2:** Distribution of local-regional recurrences and metastasis by constructed molecular subtype

	overall	Luminal A	Luminal B	HER2+	TNBC	*P* value
Local	45	9	15	7	14	0.025
Reginal	22	7	10	2	3	0.028
LRR	62	13	24	9	16	<0.001
Metastasis	145	35	43	18	49	<0.001
Cohort	510	202	105	62	141	

In OS analysis, patients with local recurrence had a worse outcome (HR: 4.255, 95% CI: 2.713-6.672, *P* < 0.001), whereas regional recurrence trended toward a significant difference (HR: 1.974, 95% CI: 0.961-4.053, *P* = 0.064). Among patients with local-region failure, HER2-positive and TNBC patients had a shorter survival time than those with luminal types (HR: 2.356, 95% CI: 1.157-4.798, *P* = 0.018). Pathological tumour stage, pathological nodal status, Ki67 index and nuclear grade were all significantly associated with LRR-free survival rate in univariate analysis (Table 3). However, in multivariate analysis of the four covariates, pathological nodal status and ki67 index still had a significant impact on LRR-free survival.

**Table 3:** Univariate and multivariate analysis of time to locoregional recurrence

	Univariate			Multivariate		
	HR	95% CI	*P*	HR	95% CI	*P*
Age (≥50y *vs*. <50y)	1.387	0.841-2.288	0.200		-	
Menopause status (pre *vs*. post)	1.493	0.907-2.459	0.115		-	
pT (T1 *vs*. T2/3)	2.158	1.207-3.860	0.010	1.625	0.902-2.926	0.106
pN (N0/1 *vs*. N2/3)	3.489	1.950-6.242	<0.001	3.295	1.828-5.939	<0.001
MP (3/4 *vs*. 2-0)	1.594	0.968-2.623	0.067		-	
ER (positive *vs*. negative)	0.724	0.440-1.193	0.205		-	
PR (positive *vs*. negative)	0.806	0.488-1.333	0.401		-	
HER2 (positive *vs*. negative)	1.617	0.942-2.776	0.081		-	
Ki67 (≥14% *vs*. <14%)	3.105	1.884-5.119	<0.001	2.897	1.701-4.937	<0.001
nuclear grade (1/2 *vs*. 3)	2.205	1.310-3.710	0.003	1.371	0.787-2.390	0.266
LVI (positive *vs*. negative)	0.936	0.545-1.606	0.809		-	
clinical response (CR/PR *vs*. SD/PD)	1.506	0.915-2.479	0.107		-	
Chemo regimen (non-Taxel *vs* Taxel)	0.946	0.551-1.623	0.839		-	

**Figure 1:**
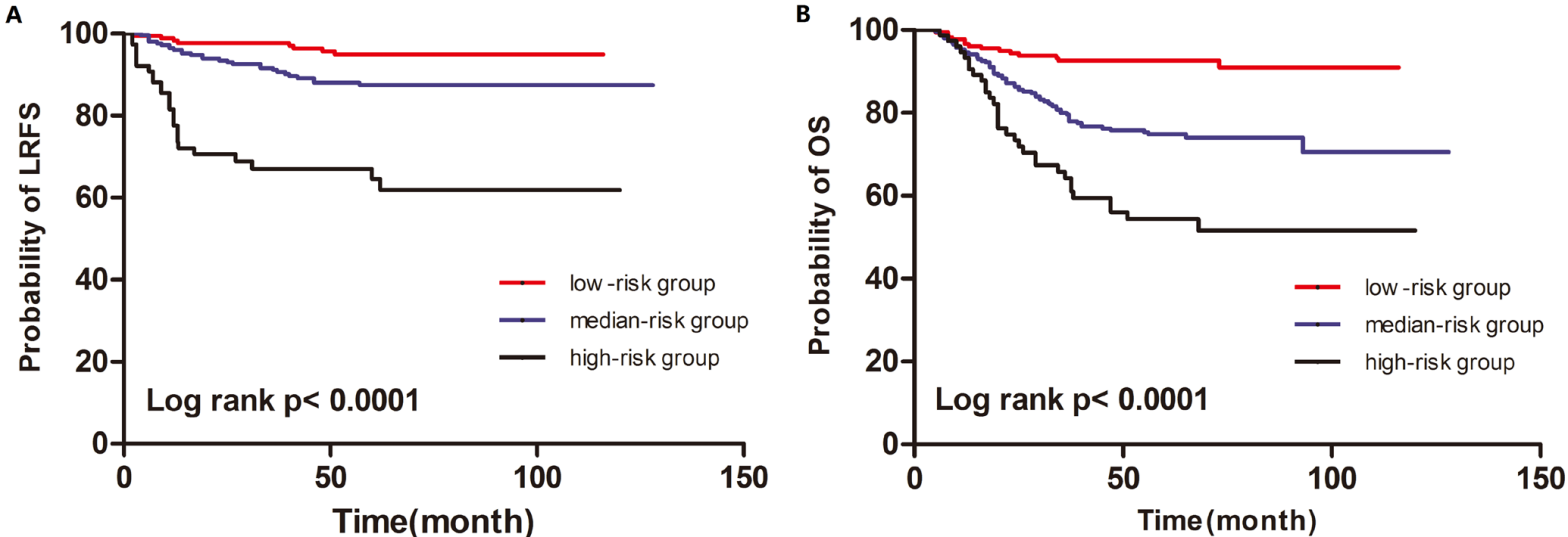
Kaplan-Meier curves for LRFS (A) and OS (B) of according to the prognostic model

After radiotherapy, 48.4% of locoregional recurrences occurred within the 1st year, 19.4% occurred within the 2^nd^ year, 11.3% occurred within the 3^rd^ year, and the remaining 21.0% of LRR occurred after 3 years. The prognostic model was established based on the sum of both independent prognostic factors, with positivity for ≥ 4 lymph nodes and Ki-67 index ≥ 14% individually contributing 1 point to the risk score. The patients were assigned to a low-risk group (0 point), median-risk group (1 point) and high-risk group (2 points). These subgroups had significantly different outcomes (Figure 1A, *P* < 0.0001 for LRFS; Figure 1B, *P* < 0.0001 for OS).

**Figure 2:**
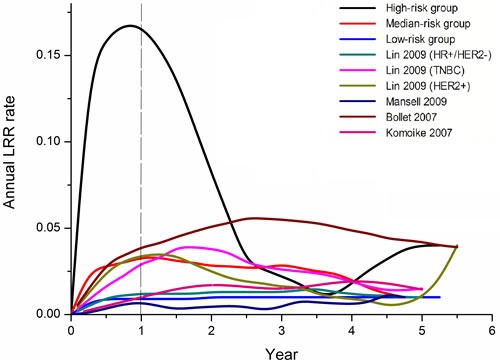
Annual recurrence rate curves derived from our data and relevant studies

The aim of the present study was to show the annual LRR rate among these 3 groups using our single-institution data, as well as to review relevant literature-based data to compare with our observations (Figure 2). Four publications that reported ARR (annual recurrence rate) after surgery for primary breast cancer were identified as eligible [17–20]. In the relevant literature, the annual risk of local-regional recurrence peaked between two and three years after the initial diagnosis. In contrast, the ARR curve of the high risk subgroup for neoadjuvant patients exhibited one peak near 1 year (17% per annum). The median and low-risk subgroups did not have an obvious recurrence peak.

## DISCUSSION

We described the rates and patterns of LRR in non-pathological complete responders who received neoadjuvant chemotherapy. In our study focusing on non-pCR patients, we excluded the pCR patients, who had the best locoregional control. Overall, patients with more advanced nodal disease and a high Ki67 index had a higher risk of loco-regional relapse. The existence of loco-regional recurrence was associated with a worse overall survival, and efforts to improve axillary control might prove of benefit in this population [5]. It remains unclear why LRR occurs after local therapy such as mastectomy and adjuvant radiation, but the results might be explained by the self-seeding hypothesis, in which it is postulated that residual cells in the breast might act as a pool of potential metastases and could seed distant sites as well as the primary tumour itself [21].

Recent publications have revealed locoregional control rates from 80% to 90% in the neoadjuvant setting, which is similar to the observation in our study [22–24]. The importance of the underlying biology of breast tumours in predicting outcome has been demonstrated by microarray analyses that identified molecular subtypes. Clinicians have used hormone receptor, HER2 status and the Ki67 index to group tumours into constructed subtypes. Patients in the luminal A subtype had the lowest LRR rate, whereas those in the luminal B subtype had the highest LRR rate. Without neoadjuvant treatment, the LRR rate after mastectomy was only 8% for luminal A tumours compared with 22% for luminal B tumours [25]. There was no clear association between the risk of loco-regional recurrence and MP stage, HR/HER2 status. Some type of breast-conserving surgical intervention is likely to be warranted, regardless of whether neoadjuvant or adjuvant treatment is adopted and regardless of the patient's initial clinical response. The local and regional recurrence rates were similar in the HR+ subgroup, whereas the aggressive subtypes such as HER2+ positive and TNBC had higher local failure rates. There is not sufficient evidence supporting this result in other reports [26]. Patients with aggressive subtypes with LRR have a shorter overall survival than those with luminal subtypes.

Although locoregional failures occurred rarely for the entire cohort, patients with involvement of more than 4 lymph nodes remained at increased risk of LRR (*p* < 0.001). The pathological status of lymph nodes was significantly related a worse relapse rate and metastasis in both the neoadjuvant and adjuvant settings [24, 25, 27]. Even after a 10-year follow-up, LRR is 16% for patients with four or more positive nodes [8]. Comprehensive nodal irradiation therapy to the full axilla might be of benefit for patients with a greater burden of residual positive disease in the axilla (≥ 4 positive nodes) [6]. However, for patients with one to three positive lymph nodes after chemotherapy, radiation did not yield a locoregional control benefit [8]. There was a very low LRR rate (1.9%) in patients who had pathologically negative lymph nodes after NCT and radiotherapy [11].

The proliferation marker Ki67 has been suggested as a promising cancer biomarker. Despite the high number of significant biomarker studies for Ki67, this marker was still used with reluctance in the clinical setting. To describe biologically the proliferative capacity of residual disease, Ki67 on the residual tumour was analysed. A total of 1150 patients from the GeparTrio study were divided into 4 groups based on different Ki67 levels, which were significantly related to DFS and OS [28]. The Ki67 cut point used in different studies varies between 5% and 34% [29, 30]. Our cut-off value is based on the 2013 St. Gallen Consensus [31]. Compared with pre-treatment measurements or changes from before and after treatment, post-treatment Ki67 was more relevant [32]. Furthermore, some research has added that post-treatment Ki67 improves the prediction of long-term outcome [33].

The rate of local recurrences was in line with those reported in the literature, which range from 12% to 30%, mostly dependent on the length of follow-up. In the adjuvant cohort, the first recurrence peak after mastectomy occurred at 2 years [34]. The annual risk of locoregional recurrence has an early peak during the first year following treatment in the neoadjuvant setting [19]. In the high-risk group, the incremental gains of locoregional control are theoretically greater. These high-risk patients might benefit most from dose-dense chemotherapy and comprehensive irradiation of the full axilla as a component of PMRT. On the other hand, novel compounds are in preparation to be assessed in the post-neoadjuvant setting for non-pCR patients. In the PENELOPE study, a novel cyclin-D kinase 4/6 inhibitor is being explored in addition to endocrine treatment. The Katherine study focuses on TDM1, which will be randomly compared with the continuation of trastuzumab. In CREATE-X trial, adjuvant capecitabine improved outcomes in women with HER2-negative breast cancer who have residual invasive disease after neoadjuvant chemotherapy. However, in the low-risk group, the annual hazard ratio was less than 0.3%. Therefore, these patients are candidates to further reduce the extent of locoregional treatment.

Our study has limitations because of its retrospective character. Furthermore, patients received different chemotherapy regimens and HER2 positive patients did not receive trastuzumab as part of the neoadjuvant treatment. Another limitation of our study is the small number of patients from which to draw meaningful conclusions about patients with luminal B/HER2- and HER2+. The appropriate identification of patients at high risk of relapse following neoadjuvant chemotherapy could enable the characterization of drivers of drug resistance [35]. High throughput technology with DNA, RNA or proteins is now available to look for a breast cancer prognostic profile. However, our nomogram could be a useful tool for predicting the risk of LRR in non-pCR responders. This would be of the utmost importance in the selection of patients who would benefit from standard treatment and those who would not. However, this nomogram requires validation in other independent data sets. Furthermore, it highlights the significance of optimizing outcomes with local and regional adjuvant therapies. The nomogram has important implications for the risk of LRR and might help to identify patients who might benefit from novel strategies to improve locoregional control. The potential is also highlighted for the design of post-neoadjuvant adjuvant studies in these high-risk populations.
